# Preoperative Glucose–Albumin Ratio and Its Association with Postoperative Outcomes in Critically Ill Adult Burn Patients: A Retrospective Cohort Study of 1119 Patients

**DOI:** 10.3390/diagnostics16152441

**Published:** 2026-08-02

**Authors:** Jihion Yu, Young-Kug Kim, Hee Yeong Kim, Yu-Gyeong Kong, Yongsoo Lee, Young Joo Seo

**Affiliations:** 1 Department of Anesthesiology and Pain Medicine, Asan Medical Center, University of Ulsan College of Medicine, Seoul 05505, Republic of Korea; jihionyu@amc.seoul.kr (J.Y.); umelly98@gmail.com (H.Y.K.); 2 Department of Anesthesiology and Pain Medicine, CHA University Gangnam Medical Center, Seoul 06135, Republic of Korea; luv000@naver.com; 3 Department of Anesthesiology and Pain Medicine, Hallym University Kangnam Sacred Heart Hospital, Seoul 07441, Republic of Korea; thisisrio@naver.com; 4 Department of Anesthesiology and Pain Medicine, Hallym University Hangang Sacred Heart Hospital, Seoul 07247, Republic of Korea

**Keywords:** blood glucose-to-serum albumin ratio, mortality, burn

## Abstract

**Background/Objectives:** Severe burn injury is associated with high postoperative morbidity and mortality due to profound metabolic and nutritional stress. The blood glucose-to-serum albumin ratio (GAR) may reflect both metabolic derangement and nutritional status, but its prognostic significance in burn intensive care unit (ICU) patients remains unclear. This study evaluated the association between preoperative GAR and postoperative outcomes in adult burn ICU patients undergoing surgery. **Methods:** We retrospectively analyzed adult burn ICU patients who underwent surgery between 2014 and 2024. GAR was calculated using blood glucose and serum albumin levels measured within one day before surgery. The primary outcome was 90-day postoperative mortality. Secondary outcomes included 90-day hospital-free days and ICU-free days. Multivariable Cox regression and restricted cubic spline analyses were performed to assess the relationship between GAR and mortality risk. Receiver operating characteristic curve analysis was used to evaluate the discriminatory performance of GAR and determine the optimal cutoff value. **Results:** Among 1119 patients, the 90-day mortality rate was 25.6%. Higher preoperative GAR was independently associated with increased 90-day mortality in multivariable Cox regression analysis. Restricted cubic spline analysis demonstrated a significant overall association, with progressively increasing mortality risk at higher GAR levels. Receiver operating characteristic curve analysis showed moderate discriminatory performance (area under the curve = 0.789), and the optimal cutoff value based on the highest Youden index was 62.5. Patients with GAR ≥ 62.5 had significantly lower 90-day survival rates and fewer 90-day hospital-free days and ICU-free days than those with lower GAR values (all *p* < 0.001). **Conclusions:** Higher preoperative GAR was significantly associated with adverse postoperative outcomes, including increased 90-day mortality and fewer hospital-free and ICU-free days in adult burn ICU patients.

## 1. Introduction

Severe burn injury is associated with substantial perioperative morbidity and mortality despite ongoing advances in surgical techniques, perioperative monitoring, and critical care management [1]. Patients with severe burns frequently develop profound hypermetabolic responses, systemic inflammation, immune dysregulation, and early organ dysfunction shortly after injury [1,2]. These pathophysiological alterations may contribute to increased postoperative complications and mortality risk. Because postoperative outcomes in critically ill burn patients may be influenced by both burn severity and underlying metabolic and inflammatory status, preoperative risk assessment may help support perioperative evaluation and clinical management in this population.

Among routinely available laboratory markers, hyperglycemia has been consistently associated with adverse outcomes in critically ill patients and may reflect stress-induced metabolic disturbance [3,4]. Hypoalbuminemia is commonly associated with persistent inflammation, increased capillary permeability, illness severity, and protein loss, while nutritional status may also contribute, particularly during the later stages of burn recovery [5,6]. In patients with severe burns, metabolic disturbances, extensive protein loss, and increased inflammation may further aggravate hyperglycemia and hypoalbuminemia during the perioperative period. Because glucose and albumin reflect distinct but complementary aspects of burn pathophysiology, integrating these biomarkers into a single composite index may provide complementary prognostic information beyond either biomarker alone. However, the prognostic utility of glucose or albumin alone may be limited in burn patients, as metabolic stress and protein depletion often coexist and interact dynamically [7,8]. Accordingly, composite measures integrating multiple physiologic domains may provide incremental prognostic information beyond individual biomarkers. One such composite biomarker is the blood glucose-to-serum albumin ratio (GAR), which has been associated with adverse outcomes across critical care and surgical populations, including sepsis [9,10], intracerebral hemorrhage [11], and major surgical procedures [12,13]. Nevertheless, the prognostic relevance of GAR has not been specifically evaluated in critically ill patients with severe burn injury, a population characterized by marked metabolic and inflammatory derangements.

Therefore, this study evaluated whether the preoperative GAR is associated with postoperative outcomes—including 90-day mortality, hospital-free days, and intensive care unit (ICU)-free days—in adult burn ICU patients.

## 2. Materials and Methods

### 2.1. Study Design and Patients

This retrospective cohort study was conducted at Hangang Sacred Heart Hospital, a tertiary university hospital affiliated with Hallym University. Adult patients aged >18 years who were admitted to the burn ICU and underwent burn surgery between 2014 and 2024 were included.

Eligibility for burn ICU admission followed institutional criteria. The criteria were as follows: total body surface area (TBSA) burned ≥20%; TBSA burned ≥10% in elderly patients (≥65 years); high-voltage electrical burns; burn injuries accompanied by significant trauma or medical comorbidities; or inhalation injury.

Patients were excluded if medical records were incomplete. Patients who were transferred to another facility after the index surgery were also excluded. For patients who underwent more than one surgical procedure during the study period, only the first operation was considered for analysis.

### 2.2. Ethical Considerations

This retrospective observational study included clinical data collected from 2014 to 2024. The study protocol received approval from the local Institutional Review Board (IRB No. 2026-004), and the requirement for written informed consent was waived due to the retrospective design. The study was performed in accordance with the ethical standards of the Declaration of Helsinki and institutional guidelines for human research.

### 2.3. Data Collection

#### 2.3.1. Data Source

Data were obtained from the clinical data warehouse of Hallym University Medical Centers. This system integrates electronic medical records, pharmacy databases, and laboratory information systems.

#### 2.3.2. Demographic and Clinical Variables

Collected variables included demographic characteristics, such as age, sex, body mass index, and American Society of Anesthesiologists (ASA) physical status, as well as medical comorbidities, including congestive heart failure, ischemic heart disease, cerebrovascular disease, diabetes mellitus, and hypertension.

#### 2.3.3. Burn-Related Variables

Burn-related data included burn type, presence of inhalation injury, TBSA burned, and post-burn days. Post-burn days were defined as the number of days from burn injury to surgery. Because burn patients may undergo dynamic metabolic and inflammatory changes over time, this variable was included to partially account for temporal heterogeneity related to the interval from injury to surgery.

#### 2.3.4. Laboratory Variables

Preoperative laboratory values, including serum creatinine, lactate, glucose, and albumin, were routinely collected within 24 h before surgery to assess baseline renal function, tissue perfusion, metabolic status, and overall physiological condition. The GAR was calculated by dividing the preoperative serum glucose concentration (mg/dL) by the preoperative serum albumin concentration (g/dL), using values obtained from the same preoperative laboratory assessment. Serum creatinine, lactate, glucose, and albumin were measured using the Alinity c Clinical Chemistry Analyzer (Abbott Laboratories, Abbott Park, IL, USA) according to the manufacturer’s instructions and standardized institutional laboratory procedures.

#### 2.3.5. Intraoperative Variables

Operative variables included operative time, vasopressor use, and red blood cell (RBC) transfusion. Operative time was defined as the duration from incision to completion of surgery. Vasopressor use was defined as any intraoperative administration of vasopressor agents.

#### 2.3.6. Definitions and Measurements

Inhalation injury was diagnosed based on bronchoscopy findings, clinical features, burn mechanism, and carboxyhemoglobin levels [14]. TBSA burned was estimated by experienced burn surgeons with more than 10 years of clinical experience. Only deep second-degree or deeper burns were included in this estimation, using a ruler-based method.

### 2.4. Endpoints

The primary outcome was 90-day postoperative mortality. Secondary outcomes included 90-day hospital-free days and ICU-free days. Hospital-free days and ICU-free days were defined as the number of days alive and out of the hospital or ICU, respectively, during the first 90 postoperative days [15]. Patients who died within 90 days were assigned zero hospital-free days and ICU-free days.

### 2.5. Burn ICU Management

All patients admitted to the burn ICU received multidisciplinary care according to institutional protocols provided by burn surgeons, intensivists, and specialized therapists [16]. Initial fluid resuscitation generally followed the Parkland formula (4 mL/kg/%TBSA burned), with additional fluids administered to maintain a target urine output of at least 0.5 mL/kg/h. Enteral nutrition was initiated within 48 h of admission when not contraindicated. Wound care consisted of routine cleansing followed by topical antimicrobial therapy and hydrofoam dressings, performed daily.

### 2.6. Intraoperative Practice

General anesthesia was administered according to institutional protocols [16]. Crystalloid solutions were infused at rates of 6–10 mL/kg/h. Synthetic colloids were administered when estimated blood loss exceeded 500 mL. RBC transfusion was initiated when hemoglobin levels decreased below 8 g/dL. Additional fluids or vasoactive agents were administered as needed to maintain a mean arterial pressure ≥ 65 mmHg.

Surgical management prioritized early excision and grafting using autografts and/or allografts. Escharotomy or fasciotomy was performed when indicated to relieve elevated interstitial pressure. In cases of suspected deep tissue infection, extensive wound debridement was followed by application of silver sulfadiazine-impregnated dressings or negative-pressure wound therapy.

### 2.7. Statistical Analyses

Continuous variables are presented as medians with interquartile ranges. Between-group comparisons were performed using the Wilcoxon rank-sum test. Categorical variables are expressed as numbers of patients and percentages and were compared using the chi-square test or Fisher’s exact test.

Multivariable Cox proportional hazards regression analysis was performed to evaluate the association between preoperative GAR and 90-day mortality. GAR was transformed using log_2_ to allow hazard ratios to be interpreted per doubling of GAR and to improve distributional symmetry and model stability. Variables considered clinically relevant or previously associated with burn outcomes in prior literature were included as candidate variables for multivariable Cox regression analysis using a forward stepwise selection approach. Multicollinearity was assessed using variance inflation factors, and variables with variance inflation factor > 10 were excluded. The proportional hazards assumption was assessed using Schoenfeld residuals. Restricted cubic spline analyses with four knots were performed to assess the association pattern between GAR and 90-day mortality.

Receiver operating characteristic (ROC) curve analysis was conducted to evaluate the predictive performance of preoperative GAR for 90-day mortality and to identify the internally derived cutoff value with the highest Youden index. Kaplan–Meier survival analysis with the log-rank test was used to compare 90-day survival between patients with GAR < 62.5 and those with GAR ≥ 62.5. All statistical analyses were performed using MedCalc (version 11.3.3.0; MedCalc Software, Mariakerke, Ghent, Belgium), R software (version 4.3.2; R Foundation for Statistical Computing, Vienna, Austria), and SPSS Statistics for Windows (version 27.0; IBM Corp., Armonk, NY, USA).

## 3. Results

**Figure 1:**
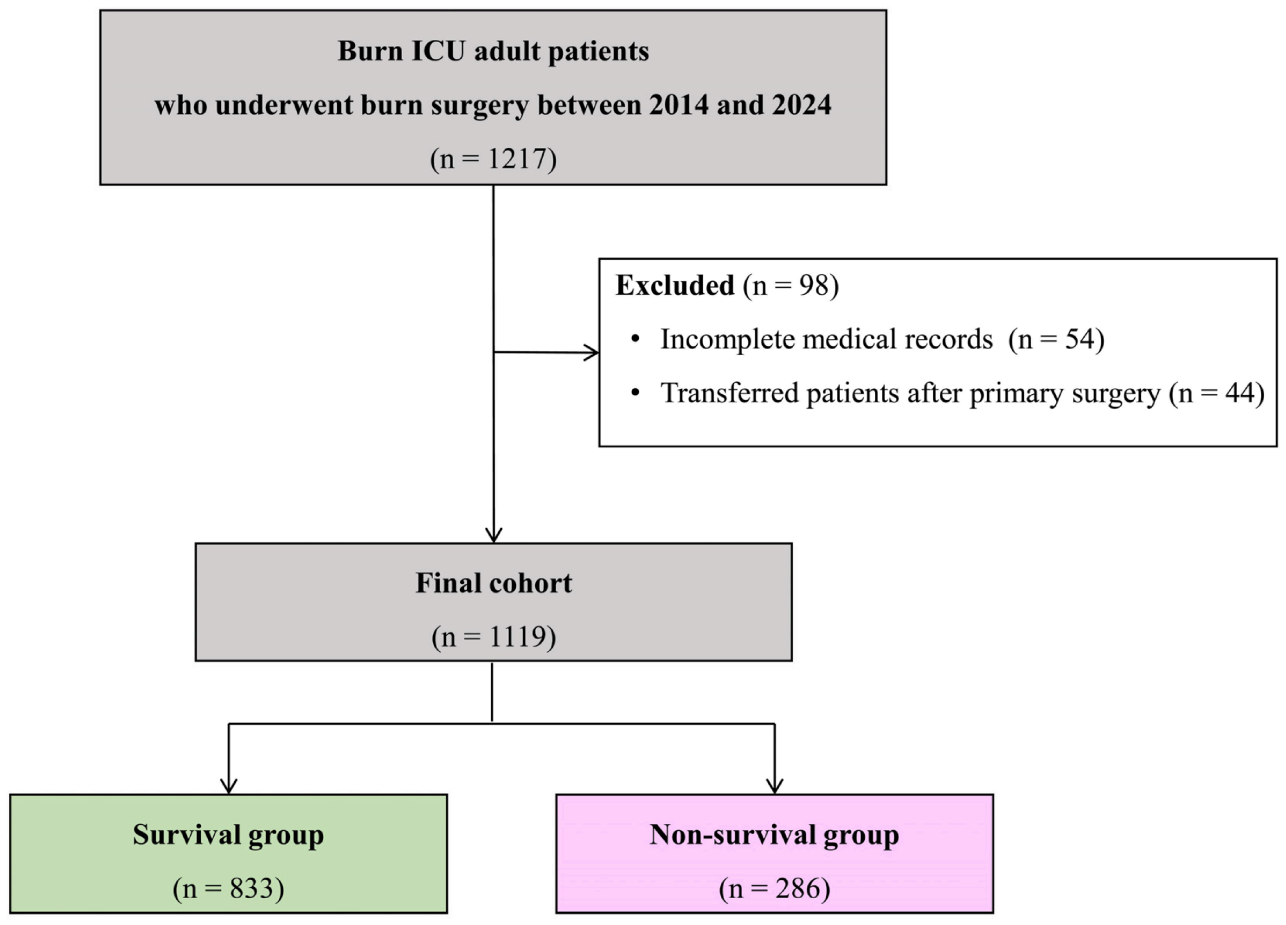
Flowchart of study patients. Patients were dichotomized into survival and non-survival groups. ICU, intensive care unit.

Among 1217 adult patients admitted to the burn ICU who underwent burn surgery between January 2014 and December 2024, 98 patients were excluded due to incomplete medical records (*n* = 54) or transfer to another hospital after the initial surgery (*n* = 44). In the incomplete medical records group, exclusions were mainly due to unclear burn history or onset timing from insufficient clinical information (*n* = 33) and missing lactate measurements (*n* = 21). After applying these exclusion criteria, a total of 1119 patients were included in the final analysis cohort (Figure 1). The overall 90-day postoperative mortality rate in the study population was 25.6%.

Baseline demographic characteristics, burn-related variables, preoperative laboratory findings, and intraoperative factors were compared according to 90-day postoperative mortality status (Table 1). Patients who died within 90 days after surgery were older and had higher ASA physical status classifications, a higher prevalence of cerebrovascular disease and diabetes mellitus, and a greater proportion of flame, scald, and contact burns than survivors. They also showed a higher incidence of inhalation injury, greater TBSA burned, shorter post-burn days, higher creatinine, lactate, and glucose levels, lower albumin levels, longer operation times, more frequent vasopressor use and RBC transfusion, and higher GAR values compared with survivors.

**Figure 2:**
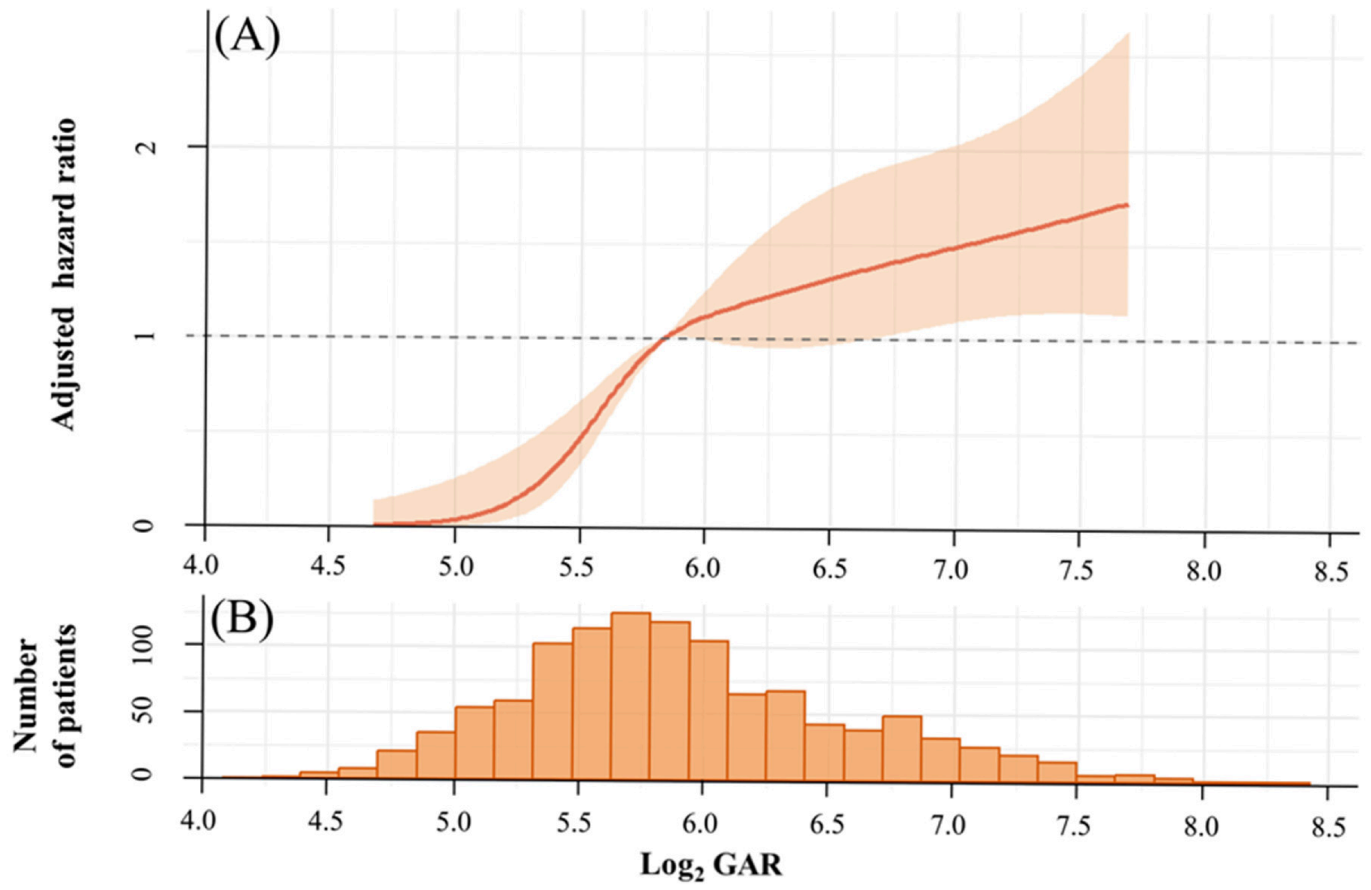
Restricted cubic spline analysis illustrating the relationship between log_2_-transformed GAR and 90-day postoperative mortality (**A**). The solid line represents the adjusted hazard ratio, and the shaded area indicates the 95% confidence interval. The histogram shows the distribution of log_2_-transformed GAR values in the study population (**B**). GAR, blood glucose-to-serum albumin ratio.

In multivariable Cox proportional hazards regression analysis, preoperative GAR, along with age, ASA physical status, burn type, inhalation injury, TBSA burned, preoperative serum creatinine, and intraoperative vasopressor use, remained significantly associated with 90-day postoperative mortality (Table 2). No significant global violation of the proportional hazards assumption was observed in the final Cox regression model using Schoenfeld residuals (global *p* = 0.120). Restricted cubic spline modeling demonstrated a significant overall association between preoperative GAR and 90-day postoperative mortality, with progressively increasing risk as GAR increased (Figure 2). Similar significant associations with 90-day mortality were also observed for admission GAR (adjusted hazard ratio, 1.575, 95% confidence interval, 1.301–1.907), preoperative GAR (adjusted hazard ratio, 1.661, 95% confidence interval, 1.385–1.993), and postoperative GAR (adjusted hazard ratio, 1.602, 95% confidence interval, 1.342–1.911) in separately adjusted models (Supplementary Table S1). Overall, GAR measured at different perioperative time points demonstrated comparable associations with 90-day mortality.

**Figure 3:**
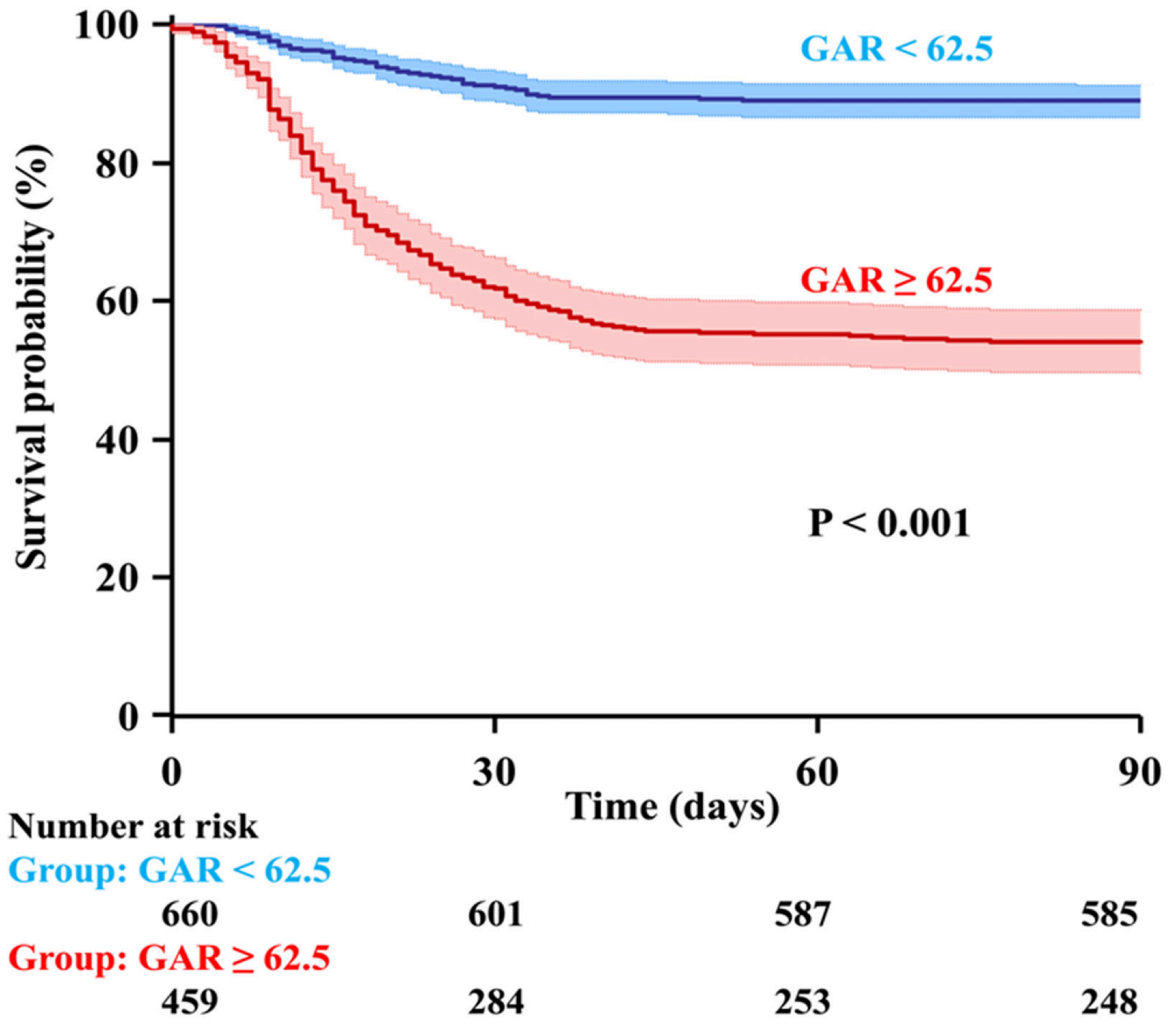
Kaplan–Meier survival analysis of 90-day mortality after burn surgery. The dashed lines indicate the 95% confidence intervals. Postoperative 90-day survival was significantly lower in patients with a preoperative GAR ≥ 62.5 than in those with a GAR < 62.5. GAR, blood glucose-to-serum albumin ratio.

ROC curve analysis demonstrated that preoperative GAR had moderate discriminatory performance for predicting 90-day postoperative mortality, with an area under the curve (AUC) of 0.789 (95% confidence interval, 0.761–0.817). The internally derived cutoff value with the highest Youden index was 62.5 (sensitivity, 74.1%; specificity, 70.4%). Compared with its individual components, preoperative GAR demonstrated significantly better discriminatory performance than preoperative glucose (AUC, 0.789 vs. 0.701; *p* < 0.001 by DeLong’s test) and preoperative albumin (AUC, 0.789 vs. 0.733; *p* < 0.001 by DeLong’s test) for predicting 90-day postoperative mortality (Supplementary Table S2). Kaplan–Meier survival analysis showed significantly lower 90-day survival in patients with GAR ≥ 62.5 than in those with GAR < 62.5 (54.0% vs. 88.6%, log-rank *p* < 0.001; Figure 3). In addition, patients with GAR ≥ 62.5 had significantly fewer hospital-free days and ICU-free days within 90 days after surgery than patients with lower GAR values, suggesting a possible association with greater postoperative illness burden and prolonged hospitalization in the high-GAR group (Table 3).

## 4. Discussion

In this study, higher preoperative GAR was significantly associated with increased 90-day postoperative mortality and was also associated with fewer 90-day hospital-free days and ICU-free days in adult burn ICU patients undergoing surgery. ROC curve analysis identified a cutoff value of 62.5 for preoperative GAR, and patients with GAR ≥ 62.5 had significantly lower survival than those with lower values. These findings indicate that preoperative GAR may reflect the patient’s overall clinical and metabolic status at the time of measurement in this retrospective cohort.

GAR is a composite measure derived from blood glucose and serum albumin levels [13]. Furthermore, GAR may provide an integrated assessment of the patient’s clinical and metabolic condition at the time of measurement. Both components are influenced by the underlying pathophysiology of severe burn injury as well as by therapeutic interventions, including glycemic management, albumin administration, and nutritional support. Therefore, GAR should be interpreted as a context-dependent prognostic indicator rather than a biologically specific biomarker, and its association with clinical outcomes should not be interpreted as evidence of a causal or mechanistic relationship. Previous studies have reported associations between GAR and clinical outcomes in several settings, including intracerebral hemorrhage [11], sepsis [9], and major surgery [12,13,17]. However, its prognostic relevance has not been specifically evaluated in adult burn ICU patients, a population characterized by substantial metabolic and inflammatory stress [1]. The observed association in our cohort may reflect the combined prognostic contribution of hyperglycemia and hypoalbuminemia. In contrast to many other clinical settings, severe burn injury is characterized by simultaneous dysregulation of glucose metabolism and serum protein homeostasis, which may increase the relevance of ratio-based markers such as GAR.

In the present study, higher preoperative GAR was significantly associated with increased 90-day postoperative mortality after multivariable adjustment. These findings suggest that GAR may reflect the combined influence of hyperglycemia [18,19] and hypoalbuminemia [20], both of which have been associated with adverse outcomes in critically ill burn patients. Restricted cubic spline analysis demonstrated a significant overall association between GAR and mortality, with increasing risk at higher GAR levels. Furthermore, higher GAR was associated with fewer hospital-free days and ICU-free days within 90 days, suggesting greater illness burden and delayed postoperative recovery. Previous studies in non-burn populations have also reported associations between elevated GAR and worse clinical outcomes [9,13,21].

We further evaluated the discriminatory performance of preoperative GAR for predicting 90-day postoperative mortality using ROC curve analysis. Preoperative GAR demonstrated significantly better discriminatory performance than either glucose or albumin alone. These findings suggest that integrating glucose and albumin into a composite index provides incremental prognostic information beyond either individual marker, supporting the use of GAR as a simple composite prognostic indicator. Furthermore, the internally derived cutoff value with the highest Youden index of GAR was 62.5. Prior studies have reported varying GAR cutoff values across different clinical settings [9,10,13,17,22]. Lower thresholds such as 0.175 [22] and 4.351 [17] were reported in neurological cohorts using International System of Units, whereas higher values such as 27.3 [10], 31.79 [13], and 47.3 [9] were reported in studies using conventional units or involving populations with greater metabolic stress. The cutoff value of GAR identified in this study was derived using ROC curve analysis and therefore reflects the characteristics of our specific study population. Previous studies have reported varying cutoff values, likely due to differences in patient populations, disease severity, timing of measurement, and clinical settings. This variability suggests that GAR thresholds are inherently context-dependent rather than universal. Therefore, the cutoff proposed in our study should be interpreted with caution and considered as a reference within similar clinical contexts rather than a definitive clinical threshold. Further external validation in independent cohorts is required to determine the generalizability and clinical applicability of this cutoff value.

In interpreting GAR in burn patients, the timing of laboratory measurement is an important consideration. Unlike general surgical populations, burn patients undergo profound and evolving metabolic and inflammatory responses over time, including the initial resuscitation phase, the subsequent hypermetabolic state, and potential complications such as infection or sepsis [1,2]. These dynamic physiological changes can substantially influence both glucose and albumin levels, and therefore directly affect the biological interpretation of GAR. In particular, during the early post-burn period, hypoalbuminemia is predominantly attributable to increased capillary permeability and systemic inflammatory responses rather than nutritional depletion alone, which should be considered when interpreting GAR. Accordingly, the biological interpretation of GAR is likely to vary according to the timing of measurement during the clinical course of burn injury. In our study, laboratory values were obtained within 24 h prior to surgery to reflect the immediate preoperative condition. To partially account for the temporal heterogeneity inherent to burn injury, post-burn day was included as a covariate in the multivariable Cox proportional hazards model. In addition, GAR was evaluated at multiple perioperative time points (admission, preoperative, and postoperative) to assess the consistency of its association with postoperative mortality across different stages of clinical care. Although post-burn day was not independently associated with 90-day postoperative mortality after multivariable adjustment, GAR showed consistent associations with mortality across all evaluated perioperative time points. Nevertheless, future prospective studies incorporating serial measurements across different phases of burn injury are warranted to better characterize the temporal changes in GAR and determine the optimal timing for prognostic assessment.

This study has certain limitations that should be acknowledged. First, exclusion of patients with incomplete records or transfer to another hospital may have introduced selection bias because complete-case analysis was used. In addition, because only patients who survived to undergo surgery were included, survival (immortal time) bias may have been introduced. Consequently, the present findings may not be generalizable to patients who died before surgical intervention, particularly during the early resuscitation period after burn injury. Second, although forward stepwise selection was used to reduce excessive model complexity in the multivariable Cox regression analysis, stepwise procedures may still be susceptible to model instability and overfitting. Therefore, the present findings should be interpreted with caution. Third, GAR likely reflects a combination of endogenous physiological responses and treatment-related factors. Although perioperative glycemic management at our institution followed a standardized insulin protocol, this retrospective study could not fully account for all treatment-related variables, including insulin administration, albumin supplementation, and nutritional support. Consequently, the biological interpretation of GAR remains limited, and residual confounding related to treatment practices cannot be completely excluded. Therefore, the observed associations should be interpreted as reflecting the overall clinical context rather than evidence of a causal or mechanistic relationship. Fourth, although intraoperative RBC transfusion was included in the multivariable Cox proportional hazards analysis, transfusion requirements and decisions are influenced by surgical complexity, intraoperative blood loss, patient condition, and institutional transfusion practices. Therefore, residual confounding related to surgical characteristics and transfusion management cannot be completely excluded. Despite these limitations, preoperative GAR remained associated with postoperative mortality after adjustment for multiple clinically relevant variables. Accordingly, GAR should be regarded as a simple adjunctive prognostic marker for perioperative risk stratification rather than a standalone or biologically specific biomarker. Furthermore, because this was a single-center study conducted in a specialized burn ICU with standardized perioperative management protocols, the present findings should be interpreted within this specific clinical context. External validation in multicenter cohorts with different patient populations and treatment protocols is required before the results can be generalized or broadly applied in clinical practice.

## 5. Conclusions

Among adult burn ICU patients undergoing surgery, elevated preoperative GAR (≥62.5) was independently associated with increased 90-day postoperative mortality, as well as fewer hospital-free days and ICU-free days. Because GAR reflects a combination of physiological and treatment-related factors, it should be regarded as a simple adjunctive prognostic marker for perioperative risk stratification rather than a biologically specific biomarker. Further multicenter prospective studies and external validation are warranted to confirm the generalizability and clinical applicability of these findings.

## Figures and Tables

**Table 1:** Demographic and preoperative variables in 1119 patients with burn operations.

Variables	Total Patients (*n* = 1119)	Survival Group (*n* = 833)	Non-Survival Group (*n* = 286)	*p*-Value
Age (years)	54 (44–65)	53 (42–63)	60 (49–70)	<0.001
Sex (male)	900 (80.4)	680 (81.6)	220 (76.9)	0.085
Body mass index	23.7 (21.6–25.7)	23.6 (21.6–25.7)	23.7 (21.9–25.9)	0.306
ASA physical status				<0.001
≤2	301 (26.9)	276 (33.1)	25 (8.7)	
≥3	818 (73.1)	557 (66.9)	261 (91.3)	
Congestive heart failure	17 (1.5)	13 (1.6)	4 (1.4)	>0.999
Ischemic heart disease	52 (4.6)	33 (4.0)	19 (6.6)	0.073
Cerebrovascular disease	78 (7.0)	50 (6.0)	28 (9.8)	0.043
Diabetes mellitus	142 (12.7)	92 (11.0)	50 (17.5)	0.007
Hypertension	292 (26.1)	206 (24.7)	86 (30.1)	0.086
Burn character				
Burn type				<0.001
Flame, scalding, and contact burn	974 (87.0)	692 (83.1)	282 (98.6)	
Electrical burn	145 (13.0)	141 (16.9)	4 (1.4)	
Inhalation injury	464 (41.5)	284 (34.1)	180 (62.9)	<0.001
TBSA burned	34.0 (21.0–50.0)	30.0 (20.0–41.0)	57.0 (39.2–76.0)	<0.001
Post-burn days	3.0 (2.0–5.0)	3.0 (2.0–5.0)	2.5 (2.0–4.0)	<0.001
Preoperative laboratory data				
Creatinine (mg/dL)	0.7 (0.6–1.0)	0.7 (0.6–0.9)	0.9 (0.7–1.2)	<0.001
Lactate (mmol/L)	2.4 (1.4–3.9)	2.2 (1.3–3.3)	3.7 (2.1–5.5)	<0.001
Glucose (mg/dL)	143 (120–177)	136 (115–163)	169 (135–217)	<0.001
Albumin (g/dL)	2.5 (2.1–2.9)	2.6 (2.3–3.0)	2.1 (1.8–2.6)	<0.001
GAR	57.0 (44.4–78.8)	51.9 (41.2–66.0)	79.8 (61.6–115.0)	<0.001
Operation				
Operation time (min)	85.0 (60.0–115.0)	80.0 (50.0–110.0)	90.0 (65.0–120.0)	<0.001
Vasopressor usage	478 (42.7)	281 (33.7)	197 (68.9)	<0.001
RBC transfusion (units)	3.0 (2.0–5.0)	3.0 (2.0–4.0)	4.0 (3.0–6.0)	<0.001

Continuous variables are described using median and interquartile ranges. Categorical variables are presented as numbers and percentages. Preoperative laboratory data were measured within 24 h before surgery. Post-burn days were defined as the number of days from burn injury to surgery. ASA, American Society of Anesthesiologists; TBSA, total body surface area; GAR, blood glucose-to-serum albumin ratio; RBC, red blood cell.

**Table 2:** Cox proportional hazards analyses of risk factors for 90-day mortality after burn surgery in 1119 patients.

Variables	Univariable Analysis		Multivariable Analysis	
	HR (95% CI)	*p*-Value	HR (95% CI)	*p*-Value
Age	1.020 (1.013–1.030)	<0.001	1.047 (1.037–1.057)	<0.001
Sex (male)	0.786 (0.597–1.032)	0.086		
Body mass index	1.021 (0.988–1.055)	0.223		
ASA physical status		<0.001		0.001
≤2	1.0		1.0	
≥3	4.495 (2.981–6.776)		2.005 (1.319–3.048)	
Cerebrovascular disease	1.492 (1.010–2.203)	0.044		
Diabetes mellitus	1.524 (1.123–2.068)	0.007		
Hypertension	1.246 (0.968–1.605)	0.088		
Burn character				
Burn type		<0.001		0.035
Flame, scalding, and contact burn	1.0		1.0	
Electrical burn	0.081 (0.030–0.218)		0.342 (0.126–0.926)	
Inhalation injury	2.821 (2.219–3.587)	<0.001	2.054 (1.595–2.646)	<0.001
TBSA burned	1.049 (1.044–1.055)	<0.001	1.045 (1.039–1.052)	<0.001
Post-burn days	0.931 (0.897–0.968)	<0.001		
Preoperative laboratory data				
Creatinine	1.659 (1.470–1.873)	<0.001	1.723 (1.459–2.033)	<0.001
Lactate	1.207 (1.168–1.248)	<0.001		
GAR	3.354 (2.867–3.924)	<0.001	1.661 (1.385–1.993)	<0.001
Operation				
Operation time	1.002 (0.999–1.004)	0.130		
Vasopressor usage	3.603 (2.804–4.629)	<0.001	1.831 (1.411–2.378)	<0.001
RBC transfusion	4.273 (2.339–7.807)	<0.001		

Post-burn days were defined as the number of days from burn injury to surgery. GAR was log2-transformed to facilitate clinical interpretability and model stability. HR, hazard ratio; CI, confidence interval; ASA, American Society of Anesthesiologists; TBSA, total body surface area; GAR, blood glucose-to-serum albumin ratio; RBC, red blood cell.

**Table 3:** Comparison of 90-day hospital-free days and ICU-free days according to preoperative GAR.

Outcomes	GAR < 62.5 (*n* = 660)	GAR ≥ 62.5 (*n* = 459)	*p*-Value
90-day hospital-free days	26.0 (0.0–44.2)	0.0 (0.0–22.0)	<0.001
90-day ICU-free days	71.0 (52.0–83.0)	11.0 (0.0–60.0)	<0.001

Data are presented as median and interquartile ranges. ICU, intensive care unit; GAR, blood glucose-to-serum albumin ratio.

## Data Availability

The data presented in this study are available on request from the corresponding authors. The data are not publicly available due to ethical restrictions.

## Supplementary Materials

The following supporting information can be downloaded at: https://www.mdpi.com/article/10.3390/diagnostics16152441/s1, Table S1: Adjusted associations between GAR at three perioperative time points and 90-day mortality; Table S2: Comparison of the prognostic performance of preoperative GAR, preoperative glucose, and preoperative albumin for predicting 90-day postoperative mortality.

## Abbreviations

The following abbreviations are used in this manuscript:

GAR	blood glucose-to-serum albumin ratio
ICU	intensive care unit
TBSA	total body surface area
RBC	red blood cell
ROC	receiver operating characteristic
ASA	American Society of Anesthesiologists
HR	hazard ratio
CI	confidence interval
AUC	area under the curve
