# Supplementary material for: Preoperative Glucose–Albumin Ratio and Its Association with Postoperative Outcomes in Critically Ill Adult Burn Patients: A Retrospective Cohort Study of 1119 Patients
Source: Diagnostics (Basel). 2026 Aug 2;16(15):2441. doi: 10.3390/diagnostics16152441 (PMC13464390; doi:10.3390/diagnostics16152441)
Supplement: Supplementary file 1 [file diagnostics-16-02441-s001.zip › diagnostics-4427880-supplementary.pdf]

Supplementary Table S1. Adjusted associations between GAR at three perioperative time points and 90-day mortality

GAR measurement timing	Adjusted HR (95% CI)	P-value
Admission GAR	1.575 (1.301–1.907)	< 0.001
Preoperative GAR	1.661 (1.385–1.993)	< 0.001
Postoperative GAR	1.602 (1.342–1.911)	< 0.001

Each model was adjusted for age, American Society of Anesthesiologists physical status, burn type, inhalation injury, total body surface area burned, post-burn days, diabetes mellitus, creatinine, and vasopressor use. Hazard ratios (HRs) are expressed per doubling of the GAR. Global P-values for the proportional hazards assumption were derived from Schoenfeld residual tests. GAR, blood glucose-to-serum albumin ratio; CI, confidence interval.

Supplementary Table S2. Comparison of the prognostic performance of preoperative GAR, preoperative glucose, and preoperative albumin for predicting 90-day postoperative mortality

Variables	AUC (95% CI)	ΔAUC vs. GAR	P-value
Preoperative GAR	0.789 (0.761–0.817)	Reference	—
Preoperative glucose	0.701 (0.666–0.735)	−0.088	< 0.001
Preoperative albumin	0.733 (0.699–0.768)	−0.056	< 0.001

Areas under the curve (AUCs) were estimated using receiver operating characteristic (ROC) curve analysis. P-values represent comparisons of the AUC for preoperative GAR with those for preoperative glucose and preoperative albumin using DeLong's test for correlated ROC curves. ΔAUC indicates the absolute difference in AUC relative to preoperative GAR. GAR, blood glucose–to–serum albumin ratio; CI, confidence interval.
